# Securing the 6G–IoT Environment: A Framework for Enhancing Transparency in Artificial Intelligence Decision-Making Through Explainable Artificial Intelligence †

**DOI:** 10.3390/s25030854

**Published:** 2025-01-30

**Authors:** Navneet Kaur, Lav Gupta

**Affiliations:** Department of Computer Science, University of Missouri, St. Louis, MO 63121, USA; nk62v@umsystem.edu

**Keywords:** IoT security, artificial intelligence, 6G networks, XAI, SHAP, LIME, 6G security, machine learning, intrusion detection, network security

## Abstract

Wireless communication advancements have significantly improved connectivity and user experience with each generation. The recent release of the framework M.2160 for the upcoming sixth generation (6G or IMT-2030) cellular wireless standard by ITU-R has significantly heightened expectations, particularly for Internet of Things (IoT) driven use cases. However, this progress introduces significant security risks, as technologies like O-RAN, terahertz communication, and native AI pose threats such as eavesdropping, supply chain vulnerabilities, model poisoning, and adversarial attacks. The increased exposure of sensitive data in 6G applications further intensifies these challenges. This necessitates a concerted effort from stakeholders including ITU-R, 3GPP, ETSI, OEMs and researchers to embed security and resilience as core components of 6G. While research is advancing, establishing a comprehensive security framework remains a significant challenge. To address these evolving threats, our research proposes a dynamic security framework that emphasizes the integration of explainable AI (XAI) techniques like SHAP and LIME with advanced machine learning models to enhance decision-making transparency, improve security in complex 6G environments, and ensure effective detection and mitigation of emerging cyber threats. By refining model accuracy and ensuring alignment through recursive feature elimination and consistent cross-validation, our approach strengthens the overall security posture of the IoT–6G ecosystem, making it more resilient to adversarial attacks and other vulnerabilities.

## 1. Introduction

Wireless communication advances from 3G to 5G have had an enormous effect on user experience, connection, and technology [1]. However, these progressions have also led to significant security challenges [2]. Despite the security advancements in 5G networks, approximately 70% of 5G operators have seen several security breaches, resulting in network outages, data leaks, and significant financial losses, according to a GlobalData research study conducted for Nokia [3]. Operators continue to report that their defenses lack the ability to address the new threats, almost five years after the initial 5G launch [4]. Drawing from experiences with earlier generations, it is expected that 6G will bring even more complex challenges for network operators. The introduction of advanced technologies in 6G could create new vulnerabilities, which cybercriminals may exploit. Moreover, the integration of massive IoT with 6G poses additional risks, as it significantly enlarges the attack surface, thereby complicating the security of an already intricate and widespread network infrastructure [5].

The ITU-R M.2160 framework’s description of the integration of sophisticated technologies like terahertz (THz) transmission, Open Radio Access Networks (O-RANs), disaggregated Het-clouds, and native AI within 6G may create new security flaws that hackers might take advantage of [6]. For instance, the switch to O-RANs expands the attack surface while encouraging innovation by disassembling conventional network components into modular structures. Substantial supply chain risks are posed by this modular architecture, including the likelihood of compromised software and hardware from multiple manufacturers, irregular adherence to security standards, and vulnerabilities from integrations by third parties [7]. The combination of sensing and communication capabilities in IMT-2030, alongside AI, is expected to support new applications like digital twins, industrial automation, and personal health, which will pose significant security challenges [8]. While terahertz (THz) communication offers high data speeds and low latency, it is susceptible to denial-of-service attacks, eavesdropping, and signal interference [9]. Similarly, transmissions might be rerouted using Intelligent Reflecting Surfaces (IRSs), which can strengthen signals, disrupt communications, or permit man-in-the-middle attacks [10]. Massive MIMO (Multiple-Input Multiple-Output) technology improves network capacity, yet it additionally presents complicated safety issues, namely, pilot contamination and challenges in safeguarding multiple antennas [11]. The growing reliance on AI within 6G networks enhances performance optimization but raises concerns about algorithm manipulation and adversarial attacks [5]. Additionally, the use of disaggregated heterogeneous clouds increases the risk of cross-tenant attacks, insider threats, and vulnerabilities tied to virtualization [12]. Lastly, integrating native AI could introduce risks like model poisoning, adversarial attacks, and AI-generated malware [8].

Anticipated 6G use cases, like collaborative robotics, immersive communication (augmented and virtual reality), and native AI integration, are expected to exacerbate existing security risks and introduce new vulnerabilities. These innovations could expose sensitive biometric data, create opportunities for content manipulation, and lead to operational threats such as command injection and denial of service (DoS) attacks. AR/VR technologies are especially vulnerable to exploitation through data manipulation, where attackers could alter or inject sensory data, potentially causing severe harm to users’ well-being [13]. Likewise, operational outages, hijacking, and other hacks could affect the performance of collaborative robotics, which depends on real-time interaction and coordination [14]. The integration of native AI into the network architecture increases the potential for misuse or manipulation, which could lead to unforeseen security breaches and unchecked proliferation of fraudulent activity throughout the network [5]. These technologies’ intricacy and continually evolving nature stresses the need for comprehensive and adaptable precautions to defend against both present and future threats.

Although the incorporation of AI into various aspects of 6G presents substantial risks, it also offers significant potential to enhance and secure new use cases. However, this potential is accompanied by challenges that need to be addressed for AI to fulfill its promise effectively. The primary challenge arises from the inherent lack of transparency in AI decision-making processes, which may erode confidence. This confidence is crucial for the successful deployment of advanced IoT–6G applications [15]. Transparency within security systems is vital for understanding how decisions are made and ensuring the fairness of security protocols [16]. Users find it hard to independently verify threat assessments as well as comprehend the rationale behind security measures, given that they lack clear visibility into AI algorithms [17]. The absence of transparency not only hampers accountability and collaboration but also heightens the risk of overlooking critical threats or misinterpreting security alerts [18].

Incorporating AI within transparent frameworks while maintaining strict human oversight is crucial for effectively addressing these imminent problems and safeguarding the emerging 6G and IoT ecosystem [19]. A significant method to boost transparency in security-related decision-making in IoT and 6G environments involves using explainable AI (XAI). By establishing the role of AI in security procedures and ensuring accountability for any breaches or failures, XAI helps security professionals comprehend and manage the dangers connected to AI-driven decisions [20,21]. The broader safety framework of the 6G and IoT ecosystem is reinforced by this transparency, which boosts confidence in AI systems and strengthens the efficacy of security measures [17]. By adopting XAI, organizations can ensure that AI technologies positively contribute to security, enhancing the resilience and reliability of next-generation communications.

Several significant problems remain despite the notable accomplishments described in past studies, as presented in Section 2. It is observed that new datasets, that can significantly improve security evaluations in actual IoT contexts, are often not being utilized [22]. Due to their propensity for imbalance or lack of balance across subcategories within classes, these datasets frequently provide skewed distributions and inaccurate forecasts [23]. Not all of the benefits of feature refinement have been thoroughly explored. With the help of XAI insights, models could be refined and accuracy improved [24,25]. Furthermore, thorough research comparing model predictions with the knowledge gained from XAI techniques is noticeably lacking as far as confirming the accuracy and consistency of forecasts is concerned. Lastly, there are still a lot of gaps in the thorough assessment and verification of outcomes from various XAI approaches. Our study is primarily motivated by the need to address these issues in order to create secure and effective IoT security solutions.

This study addresses the difficulties in 6G networks by implementing practical techniques to improve security in novel ways. Although the methods are well-established, their customized application to the intricate and changing requirements of 6G and IoT contexts presents a fresh viewpoint. We hope to build a more robust and flexible security framework that tackles particular security flaws and undiscovered threats by combining these tactics with the most recent advancements in network technology. First, we utilize comprehensive datasets that cover a broad spectrum of IoT attacks, incorporating data from over a hundred IoT devices. This dataset captures the complexity and variety of traffic patterns typically encountered in 5G and is expected to persist in 6G networks, providing a solid foundation for machine learning (ML) classifiers [22]. This diverse dataset helps the classifiers better adapt to the dynamic and multifaceted nature of IoT environments, ensuring more accurate and reliable threat detection in next-generation network infrastructures. Second, we implement robust data balancing techniques to ensure that all attack subcategories are sufficiently represented within the training dataset, improving the model’s ability to learn and generalize effectively across different scenarios [26].

To enhance model transparency and interpretability, our approach incorporates explainable AI (XAI) techniques, such as LIME and SHAP, which provide detailed, intuitive explanations for model predictions. These explanations help bridge the gap between complex machine learning outputs and human understanding, fostering greater trust in the system, even for stakeholders without technical expertise. In addition, we employ a robust feature elimination strategy that integrates insights from XAI techniques and feature importance scores derived from the model [27]. This process not only optimizes the model by identifying and removing redundant or less impactful features but also ensures that high-impact features are given appropriate focus. By aligning model refinement with XAI insights, we enhance the model’s accuracy while maintaining transparency in decision-making. A key innovation lies in cross-verifying predictions and high-impact features across the XAI methods. This ensures consistency and reliability in the explanations, offering a multi-faceted understanding of the model’s behavior. Unlike many existing studies (refer to Section 2.1), our approach does not stop at providing predictions; it validates the consistency of outcomes across varying scenarios taking different data instances, ensuring robustness in diverse and dynamic IoT environments. Furthermore, we carefully analyze the model’s predictions with XAI methods to ensure alignment and consistency for both benign and attack traffic samples. This cross-analysis ensures that the model behaves consistently across both types of traffic, adding another layer of trustworthiness to the system.

These advancements collectively establish a foundation for developing trustworthy, AI-powered security solutions tailored to the dynamic requirements of 6G–IoT networks. By emphasizing transparency, reliability, and adaptability, our method not only addresses current IoT security challenges but also sets a precedent for future research in explainable and effective AI-driven frameworks. In conclusion, the contributions of this paper include the following:

- Develop a robust and practical model using an advanced dataset that reflects the complexities of modern wireless networks, aiding in the prediction and mitigation of emerging threats in 6G.
- Apply the SMOTE balancing technique to correct class imbalances in the dataset and ensure fair representation of subcategories, thereby enhancing the reliability and accuracy of forecasts for any type of data.
- Provide understandable justifications for the predictions made by XAI techniques like LIME and SHAP, which improve model interpretability.
- Cross-validate the outcomes of XAI methods to increase the model’s decision-making dependability and transparency.
- Analyze the model’s predictions with the XAI method to ensure alignment and consistency for both benign and attack traffic samples.
- Refine the model’s accuracy through the feature elimination technique by leveraging insights gained from both XAI methods and the model itself.

This is the structure of the rest of the paper: A review of relevant work is given in Section 2. The security considerations in the context of 6G–IoT are examined in Section 3. The proposed method is described in depth in Section 4. Experiments and performance results of the suggested method are shown in Section 5. Lastly, the results, conclusions, and possible avenues for further study are covered in Section 6.

## 2. Past Research and the Innovations in This Work

### 2.1. Past Research

Numerous studies have effectively utilized neural networks to identify vulnerabilities in network traffic. However, a lot of these studies employ old datasets that do not account for the complexities of present-day networks, including large-scale designs and emerging avenues of attack [22]. A recent study, for instance, uses artificial neural networks (ANNs) to evaluate the combination of hybrid oracle-explainer method for intrusion detection systems (IDSs) using the CICIDS2017 dataset. While this approach offers human-understandable explanations [28], its applicability is limited due to the constraints of the dataset. Similarly, another study [29] implements an explainable AI (XAI) framework using techniques for intrusion detection on the NSL-KDD (2009) dataset, such as SHAP, LIME, CEM, ProtoDash, and BRCG, whereas [30] used the same dataset and SHAP to solve a multiclass classification issue. The study in [31] also used CIC-IDS 2017 for intrusion detection and employed LIME and SHAP for model explainability. In [32], using XAI and a decision tree algorithm, the authors utilized the KDD dataset to improve trust management in IDSs. The NSL-KDD dataset is also used in [33], which employed linear and multilayer perceptron classifiers and offered explanations through clear visuals. Despite the fact that these studies provide insightful conceptual information, their applicability is constrained by their dependence on antiquated datasets that do not take into consideration the dynamic and increasingly complicated network environments of today.

Even though some researchers have used more recent datasets, they frequently overlook how crucial it is to balance subcategories and use explainable AI (XAI) methodologies to make sense of the choices their AI models make. In [34], for instance, the author assessed how well different deep learning models detect cybersecurity threats in IoT networks, while in [35], machine learning techniques are used. to identify botnet activities in IoT environments. However, neither of these studies integrates XAI techniques or addresses dataset balancing, which can introduce bias and reduce predictive accuracy. Similarly, other researchers have suggested light weight deep learning techniques for detecting intrusion and DDoS attacks in IoT settings [36,37]. Additionally, the research in [38,39] explores tree-based algorithms for binary classification of malicious IoT traffic. However, their approaches also overlook data balancing and/or XAI techniques, which limits transparency and interpretability. The overall efficacy and dependability of the security solutions may be compromised by these flaws, which may cause AI models to exhibit inconsistent performance across several subcategories.

To improve the relevance and applicability of the findings in this domain, it is imperative that future studies prioritize the use of current datasets, incorporate XAI techniques, and address dataset balancing. By focusing on these critical factors, researchers can improve model transparency, reduce potential biases, and align their findings more closely with the complexities of modern network scenarios. This all-encompassing strategy will greatly increase the dependability and efficiency of AI-driven cybersecurity solutions.

While some studies interpret their findings using explainable AI (XAI), they frequently fail to consider the usage of recursive feature elimination (RFE) to iteratively improve accuracy and overall performance [25]. In the work in [40], for instance, machine learning models’ categorization judgments on NetFlow feature sets, such as BoT-IoT and ToN-IoT, are interpreted using SHAP. While this approach enhances detection accuracy, it does not incorporate feature elimination for further optimization, which could enhance model performance. Similar to this, the study in [41] uses techniques like LIME and Counterfactual XAI while emphasizing explainability in IoT intrusion detection, utilizing recent datasets like CICIoT2023 and IoTID20. However, it fails to adequately address dataset balancing and feature elimination, compromising both the accuracy and fairness of predictions.

Finally, a common limitation across many studies deploying XAI is the reliance on a single explanation method, which restricts the depth and reliability of insights. To balance the dataset and attain a 96.25% detection accuracy, the authors in [42], for example, used the SMOTE technique in conjunction with decision trees, random forests, and SVM algorithms in their intrusion detection system. However, they solely depend on LIME for explainability, omitting feature elimination techniques that could further improve model performance. In a similar vein, the authors of the paper [43] employed XGBoost for network intrusion detection, attaining 93% accuracy with the NSL-KDD dataset, yet did not implement feature refinement techniques while relying exclusively on the SHAP explanation framework. In [44], the CICIDS2017 dataset is used achieving 90% accuracy, but the authors rely solely on SHAP for explaining network anomalies, which hinders adaptability to contemporary and evolving threats.

### 2.2. Novel Contributions of This Paper

**Figure 1:**
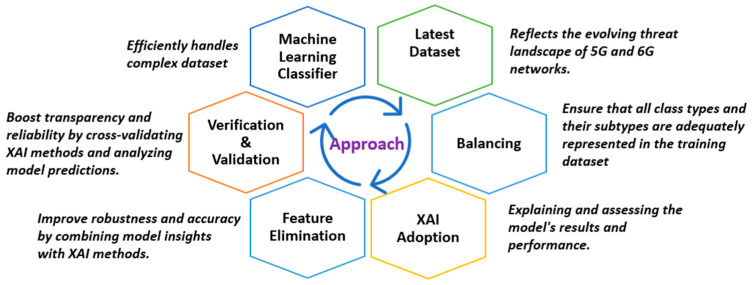
Key components of our work.

We carried out a comprehensive review of the existing body of relevant work, a summary of which is presented in Section 2.1. Figure 1 shows how our study integrates all the required elements to provide a robust explainable security framework for a 6G–IoT environment. While prior studies have explored similar areas, our work offers a fresh perspective by tailoring well-established methods to the intricate and evolving requirements of 6G and IoT environments, uniquely combining predictive capabilities for emerging IoT security threats with a strong emphasis on improving model transparency through XAI techniques. Our approach goes beyond conventional techniques by providing detailed insights into how specific feature values (high or low) influence model predictions, ensuring these features are effectively monitored for improved safety. Unlike prior studies, this paper establishes a clear and in-depth connection between the model’s decision-making process and the explanations provided by SHAP and LIME. The interpretations are presented in an accessible manner, making them understandable even to non-technical individuals or stakeholders, without prior knowledge of XAI, thus broadening the framework’s applicability and fostering trust in AI-powered security solutions.

By cross validating the outcomes of XAI methods, we increase the model’s dependability and transparency in decision-making. We also analyze the model’s predictions using XAI methods to ensure alignment and consistency for both benign and attack traffic samples, ensuring robust performance across all data instances. Additionally, we prioritize fair data representation and continuously refine predictions through validation and XAI-driven insights. This integrated approach uniquely enhances both the interpretability and effectiveness of security solutions for future networks, positioning our framework as a comprehensive, trustworthy AI-powered solution for the dynamic and complex IoT security challenges in 6G environments.

## 3. Potential Security Implications of IoT in 6G Environment

Whether the operators transition completely from 5G to 6G or start with the non-standalone implementation, the process is likely to carry over several unresolved security challenges while introducing many new ones [45]. With 6G both the complexity of the network architecture and the prolific use of IoT in the potential 6G use cases are expected to increase substantially. The developments will potentially increase threats to the IoT–6G environment in these use cases and will require attention from researchers and service providers. In this section, we describe these challenges and outline the mitigating strategies to deal with these issues. We discuss two primary categories of vulnerabilities:

1. Network- or device-level vulnerabilities
2. 6G usage scenarios-related vulnerabilities.

### 3.1. Network- or Device-Level Vulnerabilities

This section addresses the security risks associated with the network and device layers within the Internet of Things (IoT) environments. The key areas of concern include device authentication, firmware vulnerabilities, and the scalability challenges inherent in securing large-scale IoT deployments. These vulnerabilities can be exploited by attackers to gain unauthorized access or to disrupt services.

#### 3.1.1. Massive Deployment of IoT Devices

The large-scale deployment of connected IoT devices enabled by 5G greatly increases the potential attack surface. Many IoT devices suffer from inadequate security measures due to limitations in resources, including restricted processing power, memory, and battery life [45]. With 6G anticipated to support trillions of IoT devices, including ultra-low-power sensors and edge nodes, the already expansive attack surface will grow exponentially, making it harder to secure each endpoint [5]. This unprecedented growth presents new challenges for security, making it critical to develop effective security strategies.

#### 3.1.2. Weak Device Authentication and Authorization

IoT devices often rely on weak authentication mechanisms or shared credentials, making them susceptible to unauthorized access and impersonation attacks [5]. The expected increase in heterogeneous devices and autonomous systems in 6G will require the implementation of new, scalable authentication methods. Legacy devices often employ weak or compromised authentication protocols that can be easily exploited, which poses ongoing security risks [45].

#### 3.1.3. Privacy Issues in Data Transmission

IoT devices frequently collect sensitive personal or medical data, and improper encryption or insecure transmission protocols expose this data to breaches [46]. The promise of ultra-reliable low-latency communication (URLLC) and intelligent edge computing in 6G will lead to the increased processing and transmission of sensitive data, raising concerns about maintaining privacy and confidentiality at all stages of data handling [45].

#### 3.1.4. Software and Firmware Vulnerabilities

Many IoT devices operate with outdated or unpatched software and firmware, making them vulnerable to known exploits. This reliance on outdated software creates persistent backdoors that attackers can exploit [46]. Addressing this issue will be particularly challenging in the context of 6G, where ensuring timely updates across trillions of interconnected devices is crucial for maintaining security.

#### 3.1.5. Supply Chain Vulnerabilities

IoT devices are often manufactured by diverse vendors, creating difficulties in ensuring consistent security standards [5]. These devices may also contain backdoors or other vulnerabilities introduced during manufacturing [46]. As 6G advances toward broader global adoption and incorporates sophisticated IoT hardware, supply chain vulnerabilities will remain a significant risk [45].

#### 3.1.6. Lack of Standardized Security Protocols

The rapid deployment of IoT devices and networks in 5G has outpaced the development of standardized and universally accepted security protocols. The diverse and specialized use cases of 6G will amplify the need for adaptable, unified, and scalable security frameworks [22].

#### 3.1.7. AI-Related Security Issues

AI-related vulnerabilities are a growing concern in 6G networks, as AI systems are susceptible to unique attacks. These attacks can include adversarial manipulations such as digital noise or physical changes to environments that mislead AI-based systems. For instance, altering objects can confuse object recognition systems or disrupt or eavesdrop sensor data and information in IoT networks [8]. Furthermore, data poisoning can skew training sets, while model poisoning compromises AI models’ accuracy and predictions. AI-generated attacks also mimic legitimate traffic to bypass security, while privacy breaches expose or misuse sensitive data through AI systems [5].

### 3.2. 6G Usage Scenario-Related Vulnerabilities

**Figure 2:**
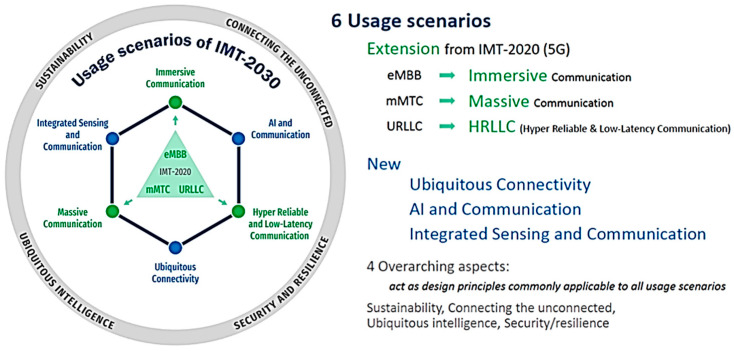
The 6 usage scenarios for 6G (source: ITU-R M.2160 [6]).

This section examines the challenges associated with the usage scenarios outlined in the ITU-R IMT-2030 framework (document ITU-R M.2160 [6]), as illustrated in Figure 2. These scenarios include both 6G enhancements to the 5G framework and new use cases specific to 6G.

#### 3.2.1. Immersive Communication

Immersive communication in 6G, including XR, holography, and telepresence, opens new possibilities for IoT devices, while simultaneously exposing them to increased security risks. The continuous exchange of personal data, including video and audio streams, raises the potential for privacy breaches, making devices susceptible to attacks like spoofing or man-in-the-middle interception [45]. The real-time nature of these systems, which requires low-latency communication, creates opportunities for attackers to disrupt critical interactions [5]. Additionally, varying network conditions—such as congestion in urban hotspots or slower connections in rural areas—can cause communication delays, signal degradation, or network outages, making devices more vulnerable to manipulation or interference [46]. These inconsistencies across different environments further complicate the security landscape of immersive communications. As immersive communication systems become more integrated into critical applications, the potential for privacy breaches, and AI-generated manipulation such as fake websites, and video or audio streams poses significant future threats leading to a broader range of security vulnerabilities, including fraud, unauthorized access, and reputational damage [13].

#### 3.2.2. Hyper Reliable and Low-Latency Communication (HRLLC)

As wireless technology moves from Ultra-Reliable Low-Latency Communications (URRLCs) of 5G to HRRLC in 6G, it poses several security challenges for IoT devices, particularly due to the stringent time constraints required for real-time applications like industrial automation and telemedicine. These stringent time constraints necessitate the prompt transmission and processing of data, which can leave systems vulnerable to security breaches that exploit the limited opportunities for security validation. For instance, malicious actors may inject harmful data during critical operational phases when delay is not an option [45]. Moreover, the focus on reliability in these applications can inadvertently lead to compromises in security measures, resulting in weak encryption protocols or insufficient access control mechanisms. The demand for precise positioning in critical applications, such as machine control and healthcare monitoring, further increases the susceptibility of IoT devices to spoofing attacks and tracking manipulations. Additionally, the high density of interconnected IoT devices in operational settings expands the potential attack surface, significantly elevating the risk of exploitation [45].

#### 3.2.3. Massive Communication

Massive communication in 6G, characterized by the interconnection of a vast number of IoT devices across diverse sectors such as smart cities, healthcare, and agriculture, presents considerable security challenges. The extensive scale of connected devices not only expands the potential attack surface but also complicates the management and security of the network [5]. Many of these IoT devices, particularly those designed for low-power or extended battery life, often lack the computational capabilities necessary to implement robust security measures, rendering them vulnerable to unauthorized data access and device manipulation [45]. Furthermore, varying network conditions—ranging from fluctuating data rates to differing mobility requirements—add another layer of complexity to the enforcement of security protocols. As these devices transition between environments characterized by low bandwidth and high-speed connectivity, inconsistencies in network performance may create critical vulnerabilities that attackers can exploit [47].

#### 3.2.4. Ubiquitous Connectivity

Ubiquitous connectivity in 6G aims to bridge the digital divide by extending access to underserved regions, yet this introduces unique security concerns [45]. The expansion of connectivity to rural, remote, and sparsely populated areas frequently necessitates interoperability with various systems and networks, which can inadvertently give rise to security vulnerabilities. IoT devices in these regions are often more susceptible to attacks due to weaker infrastructure, limited security resources, and inconsistent network reliability. Additionally, ensuring secure and seamless communication across different environments with varying network conditions exacerbates the risk of data breaches, unauthorized access, and operational disruptions [47].

#### 3.2.5. Artificial Intelligence and Communication

The integration of Artificial Intelligence (AI) with IoT in 6G gives rise to several distinct security challenges. AI-driven IoT applications, such as autonomous vehicles, medical devices, and distributed computing, rely on vast amounts of data and real-time processing, which can expose IoT devices to new vulnerabilities. IoT devices involved in distributed AI processes, such as training models or conducting inferences, are at risk of data breaches or manipulation [5]. For example, attackers may exploit weaknesses in AI models through techniques like data poisoning or adversarial attacks, leading to erroneous decision-making by the devices [45]. Additionally, the offloading of complex computational tasks across diverse networks and devices can expose sensitive data to interception or tampering during transmission, especially in scenarios requiring low latency and high reliability [47]. Privacy breaches are another significant concern, as the massive data exchange required for AI-driven applications can expose personal or sensitive information to unauthorized access [5]. The integration of AI with quantum communication and edge computing could further complicate the security landscape [2].

#### 3.2.6. Integrated Sensing and Communication

Integrated sensing and communication (ISAC) in 6G poses additional security challenges for IoT devices. These devices, which collect high-precision data for applications like navigation and health monitoring, are vulnerable to privacy breaches and data manipulation [45]. Malicious actors may attempt to falsify sensor readings, such as those related to movement or environmental conditions, resulting in incorrect system responses, particularly in high-stakes applications like fall detection or autonomous navigation [47]. Furthermore, adversarial attacks targeting the AI systems responsible for processing sensor data could significantly undermine decision-making capabilities [5]. Additionally, the integration of AI introduces new attack surfaces, enabling malicious actors to generate deceptive sensor outputs that closely mimic authentic data, thereby making detection increasingly challenging [8].

### 3.3. Mitigation Strategy

To effectively address the security vulnerabilities, XAI offers a robust mitigation strategy. XAI enhances real-time threat detection by providing transparent explanations of suspicious behaviors, which allows for faster and more precise responses to emerging threats like spoofing, data poisoning, and malicious injections [43]. This increased transparency empowers security teams to understand the reasons behind specific actions being flagged as suspicious, enabling more informed and timely responses to potential threats.

Additionally, XAI is instrumental in the analysis and interpretation of IoT device behavior and network traffic patterns. It provides clear explanations of what constitutes normal activity and identifies potential signals of an attack, thereby mitigating the risk of data breach risks in IoT devices [17]. By making the detection process more transparent, XAI helps reduce false positives, which helps the network operators extract actionable insights regarding security risks, which in turn facilitates timely interventions and reduces the time needed to address vulnerabilities [20].

In summary, XAI offers a transparent and adaptive approach to securing the 6G environment, ensuring enhanced security across the diverse and complex ecosystem of IoT devices. By prioritizing transparency and explainability, in addition to strengthening immediate threat response, XAI also contributes to the long-term resilience of security measures.

## 4. Proposed Approach

**Figure 3:**
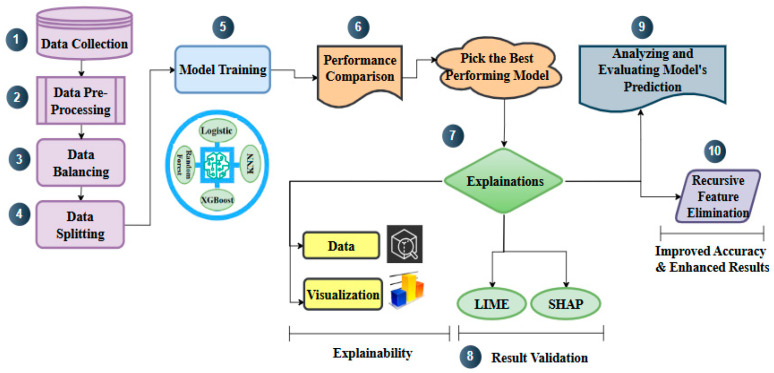
The proposed approach.

The proposed methodology outlines a systematic framework for securing IoT networks, as depicted in Figure 3. This approach follows a multi-stage process.

Initially, an appropriate IoT attack dataset is selected to train and evaluate machine learning models for classifying IoT network traffic. The second stage focuses on preprocessing the dataset by fixing null and missing values, using label encoding, and normalizing the data to ensure consistency. To rectify class imbalances and improve the model’s generalizability across different classes and subcategories, the dataset is balanced by leveraging the SMOTE technique in the third step. The dataset is divided into training and testing subgroups in the fourth step. The training dataset is used for model development and optimization, while the testing dataset is reserved for evaluating performance. In the fifth stage, machine learning models—including Logistic Regression, Random Forest, XGBoost, and K-Nearest Neighbor—are employed to classify network traffic, distinguishing between benign and attack categories. The sixth step compares the accuracy and feature important metrics of various models to assess their performance and identify the best model for additional XAI investigation. To increase transparency and offer insights into the chosen model’s decision-making process, XAI methods like LIME and SHAP are applied in the seventh stage.

The eighth stage entails cross-validating the results obtained from LIME and SHAP, ensuring transparency in the predictions. In the ninth stage, the selected or the best-performing model’s predictions are evaluated and analyzed using XAI methods, focusing on identifying and analyzing the most impactful features highlighted by them to assess their consistency and reliability. Lastly, a recursive feature removal technique is used in the tenth step to improve the model’s prediction performance and accuracy. This comprehensive framework ensures the development of a robust, transparent, and reliable security solution for both existing 5G networks and emerging 6G networks. Each step in this methodology is explained in depth in the following subsections.

Algorithm 1 outlines the pseudocode for the proposed approach. The pseudocode offers a systematic and organized depiction of the methodology used to create a robust, transparent, and reliable security solution. It details each step, including dataset selection, preprocessing, machine learning model training, the application of XAI techniques, and performance enhancement, ensuring a clear approach aligned with the goal of improving IoT network security in current and future network environments.
**Algorithm 1.** Pseudocode for the proposed approachSTART // Step 1: Dataset Selection Load CICIoT2023_attack_dataset // Step 2: Preprocessing FOR each feature IN CICIoT2023_attack_dataset: IF feature has missing/null values: Impute values using mean/median/mode ENDIF IF feature is categorical: Apply label encoding ENDIF Normalize all numerical features // Step 3: Handle Class Imbalance using SMOTE IF dataset is imbalanced: Apply SMOTE to balance classes ENDIF // Step 4: Dataset Splitting [train_set, test_set] = Split_dataset(IoT_attack_dataset, ratio = 0.8) // Step 5: Train Machine Learning Models Initialize models = [LogisticRegression, RandomForest, XGBoost, KNearestNeighbor] model_results = {} FOR model IN models: Train model on train_set Evaluate model on test_set model_results[model] = Calculate_performance_metrics(model) ENDFOR // Step 6: Compare Model Performance best_model = Select_best_model(model_results) // Step 7: Apply XAI Methods lime_explanations = Apply_LIME(best_model, test_set) shap_explanations = Apply_SHAP(best_model, test_set) // Step 8: Cross-Validate LIME and SHAP Results IF lime_explanations ≈ shap_explanations: Mark as consistent ELSE: Investigate inconsistencies ENDIF // Step 9: Analyze Feature Impact impactful_features = Identify_important_features(best_model_results,lime_explanations, shap_explanations) Validate consistency in feature rankings // Step 10: Recursive Feature Elimination WHILE model performance improves: Apply RFE(best_model, impactful_features) Retrain model Evaluate model performance ENDWHILE // Final Step: Document Results Document_results_and_framework() END

### 4.1. Dataset Overview and Collection Process

Choosing the right and appropriate dataset is essential for creating an effective security solution in IoT–6G environments. As network attacks continue to evolve, relying on outdated datasets can compromise the accuracy and relevance of detection and analysis. To address this challenge, our proposed framework utilizes the most recent CICIoT2023 dataset from the University of New Brunswick [22], which has been generated in a real-time IoT environment. This extensive dataset comprises 46,686,579 samples across 46 attributes and includes one benign class alongside 33 distinct attack types. These attacks are grouped into seven major categories: distributed denial of service (DDoS), denial of service (DoS), reconnaissance, web-based, brute-force, spoofing, and the Mirai botnet. The dataset has 169 individual CSV files, each containing a mix of benign and malicious network traffic. This provides a robust foundation for developing and evaluating comprehensive security solutions to address a wide range of threats effectively.

The 6G specifications are scheduled to evolve and get ratified by 2030. Since there are no 6G cellular implementations yet, actual operational data are not available. We have examined the ITU-R IMT-2030 framework document M.2160 [6] and believe that the CICIoT dataset reflects the environment that is expected to continue to exist in 6G deployments. It includes devices and network behaviors that are expected to be widespread in 6G, allowing us to address security challenges ahead of the full rollout of the technology. In the authors’ view, it serves as an appropriate surrogate for the environment in the upcoming cellular networks. By leveraging this dataset, we can ensure that the security measures we develop are not only relevant and scalable but also adaptable to the evolving standards of 6G, thereby laying a solid foundation for a secure 6G ecosystem.

**Figure 4:**
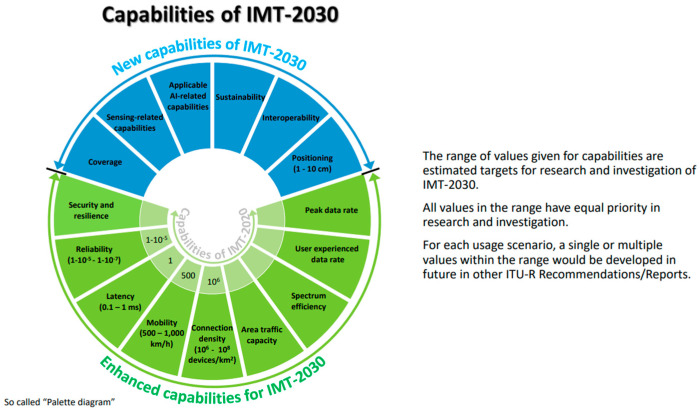
The capabilities of IMT-2030 (source: ITU-R M.2160 [6]).

Furthermore, as outlined in the ITU-R framework, 6G is intended to enhance the capabilities of 5G while integrating innovative features, as depicted in Figure 4. Since many of the technologies and features from 5G will be carried over to 6G deployments, the devices used in this dataset—such as smart home systems, cameras, sensors, smart plugs, and microcontrollers—are expected to play a key role in 6G. While these devices currently operate within 5G environments, their advanced capabilities will make them integral to the infrastructure of future 6G networks.

In this regard, the CICIoT2023 dataset serves as an essential resource for testing and validating advanced security frameworks aimed at tackling emerging threats in 6G networks. Its comprehensive structure, which aligns with the anticipated characteristics of 6G, makes it an invaluable tool for researchers and developers working to create resilient security solutions for these complex and evolving network environments [6].

### 4.2. Dataset Preprocessing

Preprocessing the dataset is a crucial step in improving and optimizing model performance. The data were initially converted into Pandas data frames [48], enabling streamlined workflows for analysis and modeling. Duplicate records were eliminated to guarantee data integrity and uniqueness, while infinite and missing values were methodically handled to reduce mistakes and biases in later modeling phases. To guarantee interoperability with machine learning algorithms, categorical labels were transformed into numerical representations. Columns with missing data were removed to maintain uniformity and reduce unnecessary complexity in the dataset. Data normalization was applied to enhance numerical consistency, improve model stability, and accelerate convergence by scaling features to a similar range. Features with zero value were eliminated, reducing the attribute count from 46 to 40, thereby focusing on the most important variables to improve model performance.

A thorough explanation of the retained features is given in Table 1. By ensuring the dataset is clean, consistent, and optimized, this thorough preprocessing approach yields model results that are more accurate and reliable.

### 4.3. Data Balancing and Data Splitting

To address class imbalances in the dataset, the Synthetic Minority Oversampling Technique (SMOTE) [26] is utilized to create synthetic samples for underrepresented classes. This method works by interpolating between an existing minority class instance $x_{i}$ and one of its *k* nearest neighbors $x_{nm}$ . The synthetic instance $x_{new}$ is generated as follows:

(1) $$x_{\mathrm{new}}=x_{i}+\lambda \cdot {(x}_{nm}-x_{i})$$

where λ represents a randomly generated number, uniformly drawn from the interval [0, 1]. This ensures that the synthetic data points are strategically placed along the line segment between $x_{i}$ and $x_{nm}$ .

Despite the variability in synthetic data points due to random selection introduced by λ , SMOTE’s ability to interpolate between data points allows for meaningful sample generation, which aids in preserving the relationships within the feature space. This allows the model to better capture the complexities of the minority class, ensuring that the synthetic samples remain relevant and contribute to the model’s improved performance in classification tasks, especially for imbalanced datasets [49].

For the next step, the problem is reframed as a binary classification task to distinguish between malicious and benign network traffic, and then the dataset is divided into training and testing sets using an 80–20 split. This stratified approach allows for a robust assessment of the model’s performance and generalizability, contributing to the development of effective security solutions.

### 4.4. Model Training

For model training, we employ four classifiers: Logistic Regression, XGBoost (Extreme Gradient Boosting), CNNs (Convolutional Neural Networks), Random Forest, and K-Nearest Neighbors (KNNs). These models were selected for their diverse approaches to classification, allowing for a comprehensive evaluation of different methodologies in detecting malicious and non-malicious network traffic.

XGBoost is a powerful and efficient supervised learning algorithm designed for both regression and classification tasks [50]. It operates based on the boosting technique, where numerous decision trees are built consecutively to improve predictive performance. Consider the dataset $D={\left\{\left(x_{i},\;y_{i}\right)\right\}}_{i=1}^{n}$ , in which $x_{i}$ represents the input characteristics and $y_{i}$ represents the associated labels. The anticipated value ${\hat{y}}_{i}$ for the *i*th instance in XGBoost is given as follows:

(2) $${\hat{y}}_{i}=\emptyset (x_{i})\sum_{k=1}^{k}{(f_{k}(x}_{i}))$$

Here, K is the total number of trees, and $f_{k}$ is the prediction from the k -th tree. $\emptyset \left(x_{i}\right)$ represents the aggregate of all individual tree predictions. This iterative training process optimizes performance by effectively minimizing errors, making XGBoost a robust tool for identifying complex patterns in network traffic data.

Random Forest serves as another ensemble learning approach that constructs numerous decision trees during training and returns the mean prediction for regression tasks [51] or the class modes for classification tasks. Let $T(x,\;\emptyset_{k})$ be the prediction given by the k -th tree. In classification, the predicted value ${\hat{y}}_{i}$ for the Random Forest is represented as follows:

(3) $${\hat{y}}_{i}=\frac{1}{K}\sum_{k=1}^{k}{(T(x,\;\emptyset_{k}}_{i})$$

Here, where the characteristics of the *k*-th tree are denoted by $\emptyset_{k}$ and the total number of trees is represented by K .

Another straightforward yet efficient classification method, the K-Nearest Neighbors (KNNs) algorithm identifies a new data point by looking at the most prevalent class among its closest neighbors in the training data [52]. After identifying the k data points closest to the new instance, the algorithm either computes an average value based on these neighbors or assigns the class label based on a majority vote. For an instance x , the predicted class $\hat{y}$ can be determined by:

(4) $$\hat{y}=\arg{max}_{y_{j}}\sum_{i\;\in \;N_{k}(x)}\;I\left(y_{i}=y_{j}\right)$$

Here, I is the indicator function, $y_{i}$ is the class of its i -th neighbor, and $N_{k}(x)$ represents the set of *k*-nearest neighbors to x .

Logistic regression is used to calculate the probability p of a binary outcome (e.g., 1 for “success” and 0 for “failure”) based on input features [53]. Mathematically, this can be represented as follows:

(5) $$p=\frac{1}{1+e^{-(\beta_{0}+\sum_{i=1}^{n}\beta_{i}x_{i})}}$$

where $\beta_{0}+\sum_{i=1}^{n}\beta_{i}x_{i}$ is the linear combination of input features. The sigmoid function maps z to the probability p between 0 and 1. If p≥0.5 , it predicts Class 1; p<0.5 , predicts Class 0.

For tabular data, Convolutional Neural Networks (CNNs) are adapted to detect patterns in structured datasets by applying filters (convolutions) to the input. While CNNs are typically used in image data, in tabular data, convolutions can be used to capture local dependencies or relationships between features. The filters slide over the data to learn feature interactions, and the network reduces the data dimensions using pooling layers before passing them through fully connected layers for final predictions [54]. The convolution operations are represented with the formula:

(6) $$y_{i,j}=\sum_{m}\sum_{n}x_{i}+m,j+n\cdot w_{m,n}$$

where x is the input data, w represents the filter (weights), y is the resulting output after the convolution operation, i,j are the indices of the output feature map, and m,n are the filter indices.

This operation allows CNNs to capture local patterns within the data, even for structured tabular data.

### 4.5. Explainability of Model Behavior

In this work, we leveraged SHAP and LIME to offer detailed explanations for the model’s predictions. SHAP is a well-known strategy for interpreting individual predictions from predictive machine learning models [55]. It computes the influence of each characteristic on given predictions using Shapley values, which are derived via cooperative game theory. The SHAP value for feature i in the context of prediction x can be defined as follows:

(7) $$\emptyset_{i}\left(x\right)=\sum S∁\left\{1,2\ldots .p\right\}\left\{i\right\}\frac{\left|S\right|!\left(p-\left|S\right|-1\right)!}{p!}\left[f\left(S\cup \left\{i\right\}\right)-f\left(S\right)\right]$$

Here, the variable p is the total number of features, the SHAP value for feature i at instance x is denoted by $\emptyset_{i}\left(x\right)$ , the model’s prediction function is given by f , S is the subset of the features excluding i , and $\left|S\right|$ indicates the number of features in subset *S*. The term $\left|S\right|!\left(p-\left|S\right|-1\right)!$ represents the number of possible ways to select the subset S .

In SHAP, the feature subsets S used in the value calculation are derived from all possible feature combinations. While the selection of these subsets introduces some level of randomness, which can cause slight errors in the final interpretation, the overall result remains stable and robust. The aggregation of contributions from all subsets reduces the impact of any single subset, ensuring that variations in feature importance estimates are minimal and do not significantly affect the overall interpretation of the model’s predictions [55].

LIME offers an alternative approach for interpreting individual predictions made by machine learning models. It functions by replicating the model’s behavior locally within a given instance, using a simpler, easier-to-comprehend model. This local approximation is obtained by minimizing a loss function $L(f,g,\;\pi_{x})$ , where the proximity measure $\pi_{x}$ defines how much weight is given to different data points based on their closeness to x [56]. The loss function is defined as follows:

(8) $$\hat{g}=\arg{min}_{g}\;L(f,g,\;\pi_{x})+\Omega \left(g\right)$$

Here, the interpretable model is denoted by $\hat{g}$ and the complexity measure of g is represented by Ω(g) .

In LIME, the weight function determines the importance of nearby points when approximating the model locally. In high-noise scenarios, the noise can distort the weighted data points, but LIME mitigates this by focusing on local areas, where the model’s behavior is most consistent. Even with noisy data, the surrogate model is designed to reflect the true model’s decision boundary, making explanations meaningful [57].

The insights provided by both SHAP and LIME enable stakeholders to better understand and trust the results of machine learning applications. These techniques significantly increase our ability to verify the model’s decision-making procedures and guarantee the accuracy of its predictions.

### 4.6. Recursive Feature Elimination

Recursive Feature Elimination (RFE) is an effective feature selection technique that we use in our study to enhance model performance [27]. RFE progressively eliminates features that are unnecessary or redundant by evaluating each attribute’s contribution to the prediction capability of the model.

Let y be the target variable and X be the feature matrix with n features. All characteristics are present at the start of the operation, i.e., $X^{\prime }=X$ . Following the training of a machine learning model with $X^{\prime }$ and y , feature significance scores are calculated using $F=\left\{f_{1\;},\;f_{2,\;},\;\ldots ,f_{n}\right\}$ .

The method then eliminates, $f_{min}=argmin\;(F)$ the least significant feature, from $X^{\prime }$ . Until the target number of features is removed or a predefined stopping condition is satisfied, this procedure is repeated. The goal is to find a smaller feature set that either keeps the performance score the same or raises it over the prior set. The following is how this procedure is represented mathematically:

(9) $${Score(X}_{new})={argmin}_{x_{new}\;}{Score(X}_{new})\;subject\;to\;{Score(X}_{new})\;\geq {Score(X}_{old})$$

By keeping just the most crucial features, RFE aims to improve model performance. By doing this, the model’s accuracy is increased, and noise is decreased. RFE helps to increase the accuracy and efficiency of the model by reducing superfluous information and concentrating on important characteristics, making sure that the elements that have the most influence on predictions are given priority.

## 5. Experiments and Results

The experiments were conducted using Python 3 in a Google Colab environment with GPU support. Several libraries and tools were used to support various workflow steps, including feature selection, interpretability, data preparation, model training, and visualization. For data manipulation and management, we utilized Pandas (version 2.2.2) and NumPy (version 1.26.4), while Scikit-learn (version 1.6.1) provided machine learning algorithms and preprocessing functions.

To develop advanced learning models, TensorFlow (version 2.17.1) and Keras (version 3.5.0) were used, offering robust frameworks for building and training neural networks. For visualizing the results, Matplotlib (version 3.10.0) and Seaborn (version 0.13.2) were employed to create clear and informative graphical representations. Additionally, SHAP and LIME were used to explain and interpret model predictions, ensuring trust and security in advanced networks like 6G, which is expected to rely on autonomous and intelligent decision-making capabilities, crucial for safeguarding critical systems and maintaining system integrity.

This integrated toolset ensures efficient data processing, reliable model development, and insightful visualizations and explanations. With the increasing complexity and demands of future 6G networks, this framework provides a scalable approach to building and optimizing security solutions.

### 5.1. Curating Data for Training Models

Processing the complete dataset is not practical due to computational constraints. For training and assessing the machine learning models, we thus arbitrarily chose 18 CSV data files from a total of 169. A total of 100,000 entries in these chosen files were utilized to train and assess the model.

Table 2 provides a description of the attack classes present in the selected files along with the benign class. The main goal of this approach is to build a transparent and reliable model utilizing XAI techniques, prioritizing interpretability and prediction clarity rather than exhaustive dataset coverage.

### 5.2. SMOTE-Based Data Balancing

**Figure 5:**
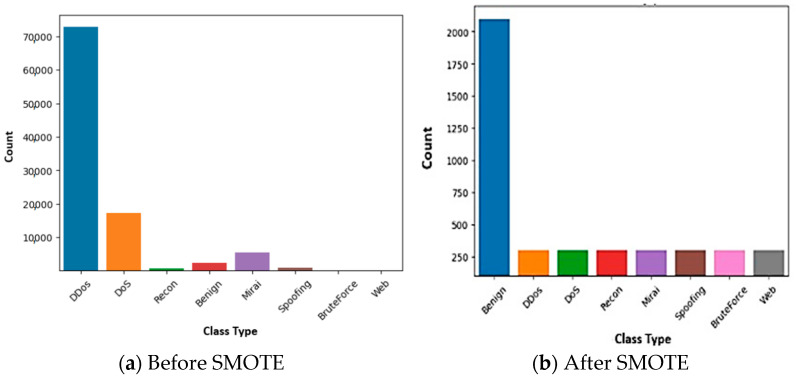
Visualization of class categories and their counts, before and after using SMOTE.

Figure 5a illustrates the unequal distribution of samples among the various categories. We ensured balanced representation by applying SMOTE to both the primary classes and their corresponding subcategories to rectify this imbalance. We standardized the number of samples in the attack categories to equal that of the benign class while maintaining dataset balance. Additionally, we made sure that every attack class subclass was suitably balanced to avoid prejudice. This approach effectively reduces the risk of the model favoring the majority classes, thereby enhancing overall performance and accuracy.

The results of the balancing exercise are illustrated in Figure 5b, which confirms the improved class distribution. Initially, the benign class, which consisted of 2376 samples (as shown in Table 2), was standardized to 2100 samples to facilitate better comparability with other classes while preserving robustness and manageability. Following the application of SMOTE, each attack subcategory was adjusted to 300 samples, achieving a total of 2100 samples across all attack classes. This balanced distribution ensures fairness across classes and enhances the model’s ability to accurately detect and classify various types of traffic.

**Figure 6:**
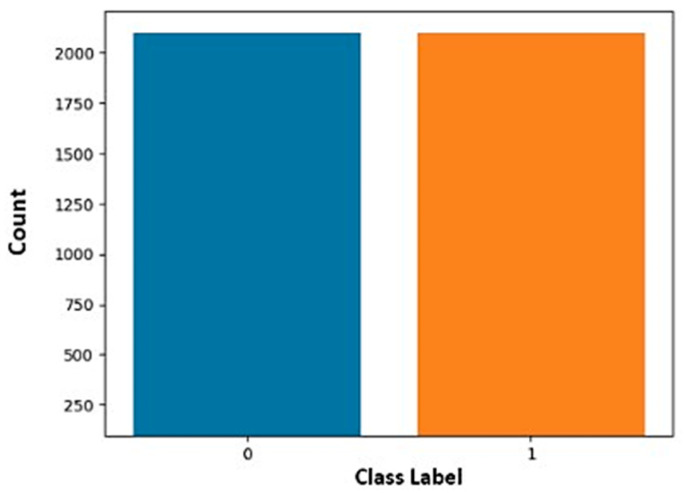
Class label distribution—post-SMOTE.

To distinguish between malicious (attack) and benign (non-malicious) network traffic, the problem was also restated as a binary classification task. This simplification focuses on two primary categories, streamlining the model’s training and evaluation process, while improving accuracy and efficiency in identifying security threats. Figure 6 further illustrates the benign (0) and attack (1) classes’ distribution.

### 5.3. Performance Evaluation of Models

In this section, we carry out model training to accurately categorize network traffic either as an “attack” or “benign”. To determine which model is best, we analyze the efficacy of several models, such as Logistic Regression, XGBoost, Random Forest, KNN, and CNN (the architecture includes dense layers with 120, 80, 40, and 20 neurons, followed by an output layer with a single neuron), using the performance criteria shown in Table 3.

Based on the analysis, XGBoost outperforms Logistic Regression, Random Forest, KNN, and CNN in terms of accuracy and other evaluation metrics. This suggests that XGBoost is more adept at handling the complexities of the dataset, making it a superior choice for classifying network traffic. While more improvement and fine-tuning of these models may be investigated to get higher accuracy, our primary focus is still on making sure that the models produce understandable outputs using XAI approaches such as SHAP and LIME. This not only aids in model validation but also builds trust in the system, especially when dealing with critical security decisions in future networks like 6G.

### 5.4. Model Selection for XAI Technique Application

In this section, we focus on selecting a machine learning model on which we can apply XAI techniques. It offers useful details regarding the model’s decision-making process for determining whether network traffic is an attack or benign. To achieve this, we compare the two highest-performing models—XGBoost and Random Forest—evaluate their consistency in predicting network traffic, and identify key features used in the classification. Both models are evaluated further by creating feature importance lists that order the features from the most to least significant and feature importance plots that highlight the value of each feature. These plots and lists identify the key features the models rely on to predict whether the traffic is benign or an attack. This analysis reveals the specific features that the models consider crucial for determining whether the traffic is benign or an attack. By comparing the models, we determine how aligned they are in their predictions. If both models use the same features for classification, we can confidently conclude that these features are indeed critical in determining the predicted outcome.

**Figure 7:**
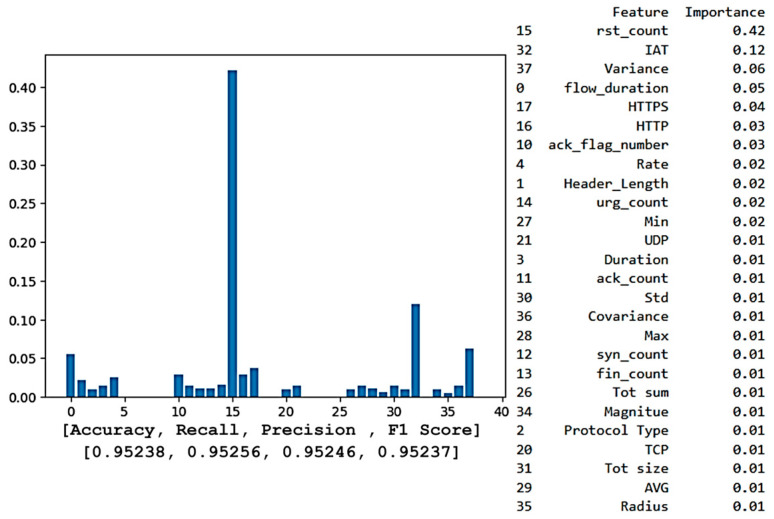
Feature importance plot for XGBoost and corresponding feature importance scores.

Figure 7 illustrates a feature importance plot for the XGBoost model, showcasing the ranking of features that significantly influence the model’s predictions. This ranking is crucial for identifying the most important features and can help in making decisions about feature retention or removal for optimal model performance. According to the plot, the most important feature that contributes to the output result is Feature 15 (rst_count), followed by Features 32 (IAT) and 37 (Variance). Features not listed in the importance ranking have a score of zero, indicating no impact on the model’s predictions, allowing for streamlined refinement.

**Figure 8:**
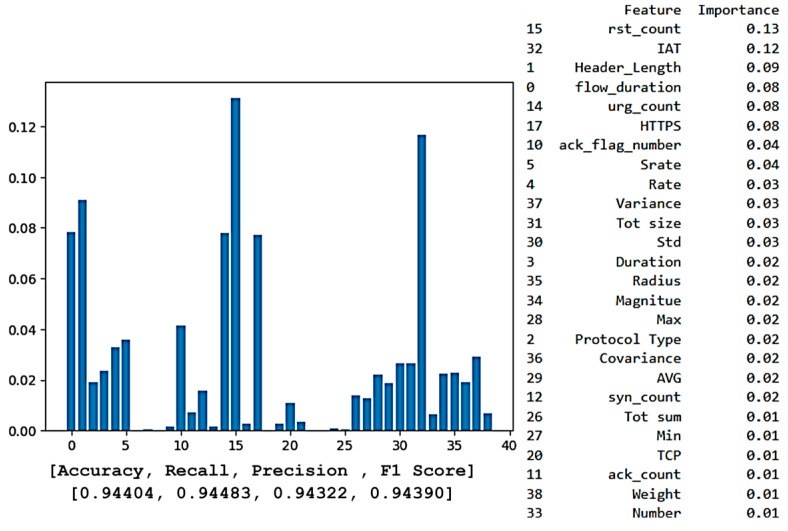
Feature importance plot for Random Forest and corresponding feature importance scores.

Despite having a slightly lower accuracy of 94.04% than the XGBoost model, the Random Forest model consistently found Feature 15 (rst_count) to be the most relevant and influential, followed by Feature 32 (IAT), as Figure 8 illustrates. The significance of these variables in reaching high accuracy levels is made apparent by their consistent prominence, which highlights the crucial role they play in the models’ predictions.

Table 4 illustrates the top six features identified by both XGBoost and Random Forest, revealing a convergence in feature importance between the two models.

The alignment between XGBoost and Random Forest emphasizes the significance of “rst_count” (the number of TCP reset packets in network traffic) in identifying patterns associated with connection resets, which are commonly linked to denial-of-service or scanning attacks. Similar to this, “IAT” is essential for identifying atypical traffic patterns, including unexpectedly long or short packet intervals. For instance, a rapid increase in IAT might be a sign of a DDoS attack, in which a network is overloaded by sending many packets at erratic intervals. An abnormally low IAT, on the other hand, might indicate a Brute-Force attack, which is defined by quick, consecutive efforts to compromise a system.

Other variables, such as “flow_duration” and “HTTPS”, are also required for detecting network traffic since they give important information about the type of communication. The “flow_duration” monitors the duration of the connection, which aids in detecting abnormalities such as abnormally short connections, and may indicate a DoS attack. The feature “HTTPS” shows whether the traffic is secure or not, as the attackers frequently target unencrypted traffic, and normal exchanges are usually encrypted. Therefore, by including these indicators, the model can better distinguish between typical and suspicious traffic, improving its capacity to detect both benign and attack network traffic.

Following this comprehensive comparison, we conclude that the model’s predictions can be deemed reliable, as two methods, XGBoost and Random Forest, identified a similar set of features for determining network traffic. To further promote transparency and explain the model’s decision-making process, we employ XAI techniques, focusing solely on the XGBoost model. This decision stems from its superior accuracy compared to the Random Forest, making it the more reliable choice for detailed analysis. Concentrating on a single, higher-performing model simplifies the explanation process and ensures that our interpretative efforts are based on the most accurate and reliable predictions, thus avoiding redundancy and maintaining clarity in our findings.

### 5.5. Enhancing Model Explainability and Transparency with SHAP and LIME

This section examines the model’s decision-making process, utilizing SHAP and LIME to improve explainability, which is especially crucial in the context of 6G networks. As 6G networks are expected to be more autonomous and complex, ensuring transparency in model predictions will be vital for maintaining security and trust. These approaches deconstruct the model’s predictions, providing a comprehensive understanding of how different features influence the final classification. By increasing transparency, we not only build confidence in the model’s predictions but also enable system administrators to make better-informed decisions in dynamic 6G environments. To explore these explanations through SHAP and LIME, we analyzed two representative test samples, referred to as Sample 1 and Sample 2, to understand the impact of specific features on the model’s classification as “Benign” or “Attack”.

#### 5.5.1. SHAP—Global Behavior Analysis

**Figure 9:**
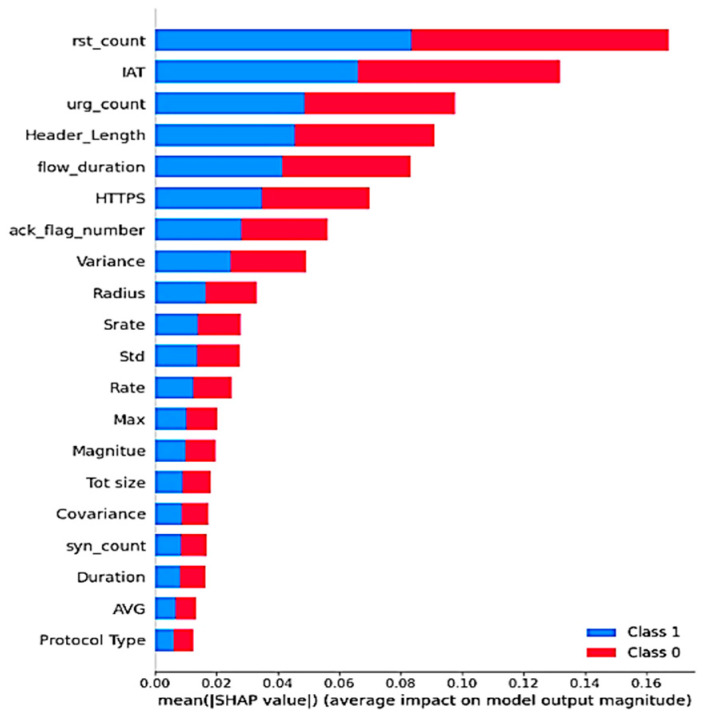
Summary plot using SHAP values and test set (Global Explanation).

We used the SHAP approach to examine the global behavior of the XGBoost model, focusing on the features that have the most significant impact on predictions across the entire dataset. Figure 9 highlights the key features identified by SHAP, ranking them based on their overall influence on the model’s predictions. The Y-axis lists these features in descending order of importance, with “rst_count” identified as the most significant and “Protocol Type” as the least influential. The X-axis indicates the mean absolute value for the SHAP method, with color coding to differentiate between the classes (0 for the Benign Class and 1 for the Attack Class). According to the findings, SHAP classifies just 20 of the 40 features as relevant and impactful, while ignoring others with little or no influence. Furthermore, the summary plot shows that the top five characteristics that substantially influence the model’s predictions are “IAT”, “rst_count”, “urg_count”, “Header Length”, and “flow_duration”.

This thorough examination of model explainability through SHAP and LIME helps to present a comprehensive understanding of feature contributions. The insights gained from this analysis are crucial for developing trust in machine learning models and ensuring that decisions informed by these models are based on transparent and interpretable metrics. By elucidating the factors driving model predictions, we contribute to improved decision-making processes for users and stakeholders alike.

#### 5.5.2. SHAP—Local Behavior Analysis

The global summary plot of SHAP provides a comprehensive overview of feature relevance over the whole dataset, indicating which factors have the most and least effect on the model’s predictions. However, it does not clarify how these attributes influence each prediction or why the model made a certain conclusion for a single data point. This constraint emphasizes the need for local analysis, which gives instance-specific knowledge of feature contributions, thereby improving the model’s interpretability and dependability.

**Figure 10:**
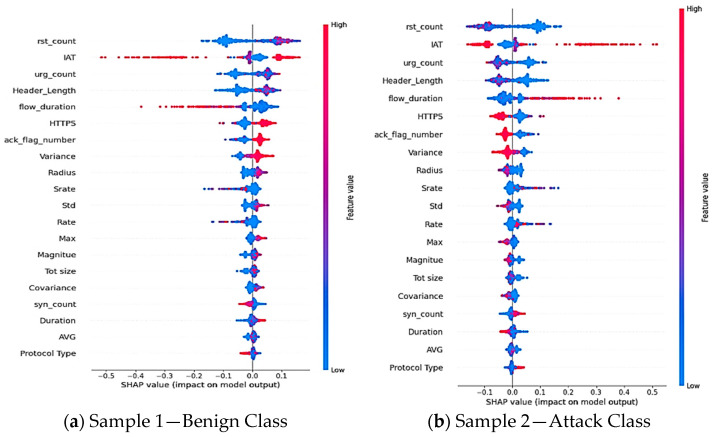
Local analysis using the SHAP summary plot: (**a**) illustration of a “Benign” class prediction for Sample 1 and (**b**) illustration of an “Attack” class prediction for Sample 2.

To demonstrate this, we use the local summary plot of SHAP to evaluate and visualize each feature contribution, to the final prediction, offering a deeper understanding of the model’s decision-making procedure for specific instances. Figure 10 depicts the SHAP local summary graphic, with red and blue hues denoting high and low feature values. Features are ordered based on their impact on the model’s output, with “rst_count” having the most impact and “Protocol Type” having the least. Figure 10a clearly illustrates a “Benign” class prediction, whereas Figure 10b clearly represents an “Attack” classification.

After analyzing the top five features from SHAP local summary plot—“rst_count”, “IAT”, “urg_count”, “Header_Length”, and “flow_duration”, the following conclusions can be drawn:

- Higher values of “rst_count” can signal abnormalities or possible attacks, whereas a low number denotes fewer network resets, which are frequently linked to benign traffic.
- Longer packet intervals, which are characteristic of benign traffic, are indicated by a high “IAT” (Inter-Arrival Time) value. On the other hand, a low number of “IAT” can indicate a pattern of unusual activity, such as DoS attacks.
- While a large value of “urg_count” can signify anomalous activity or possible attacks, a low value indicates fewer urgent packets, which is typical in innocuous traffic.
- Standard packets typically have a small value of “Header_Length”; however, a large value might suggest the existence of malicious activity or specialized protocols.
- Longer “flow duration” denotes genuine connections, whereas an infrequent one can imply possible malicious exchanges.

**Figure 11:**
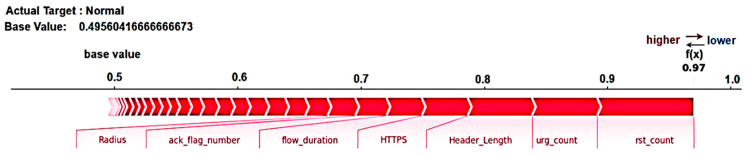
SHAP force plot for testing Sample 1 (Local Explanation)—predicting “Class 0—Benign Traffic”.

To further analyze individual test instances (Sample 1 and Sample 2), we employ SHAP force plots, which provide a detailed view of how each attribute influences a particular prediction. The plot’s base value represents the average model output across the training dataset, serving as a starting point to understand the impact of each feature on the prediction. Positive contributions are shown by red bars, while negative contributions are represented by blue bars. The length of each bar indicates the extent of the feature’s contribution and its influence, with wider bars implying a greater effect. The final prediction, shown at the end of the figure, represents the cumulative effect of all the feature’s contributions beginning from the base value. Figure 11 shows the SHAP force plot for Sample 1, and the following conclusions have been drawn from it:

- The attributes—“rst_count”, “HTTPS”, “urg_count”, “Radius”, “ack_flag_number”, “flow_duration”, and “Header_Length”—are displayed in red, collectively pushing the prediction score from the base value of 0.49 towards a higher value, thereby supporting the model’s classification of the network traffic as “Benign”.
- The red color signifies a positive contribution to the final outcome or prediction. For example, higher values of “rst_count” may point to typical session terminations rather than suspicious activities, suggesting benign traffic. Similarly, higher values of “HTTPS” are commonly linked with legitimate and secure communication, further indicating that the traffic is benign.
- A noteworthy observation here is that the SHAP force plot for this instance does not highlight “IAT”, a feature previously identified as crucial in the global model. This discrepancy underscores the importance of local explanations, as the influence of each feature can vary significantly across individual data points. It suggests that while “IAT” is generally an important feature, its impact on this specific instance is minimal.

**Figure 12:**
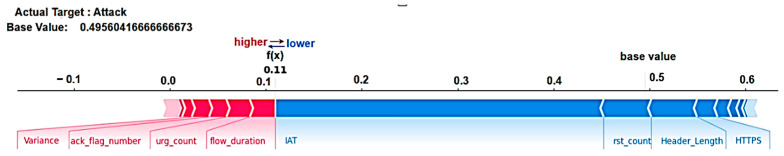
SHAP force plot for testing Sample 2 (Local Explanation)—predicting “Class 1—Attack Traffic”.

Figure 12 shows the SHAP force plot for Test Sample 2, having a base value of 0.49 and the final prediction as “Attack”. The following observations can be made:

- The “IAT” (Inter-Arrival Time) feature, represented by a lengthy blue bar, negatively influences the prediction. This negative contribution shifts the classification away from benign traffic, increasing the likelihood of identifying the traffic as an attack. Longer inter-arrival intervals between packets often indicate irregular traffic patterns, which could signify malicious activity.
- Negative contributions from features such as “rst_count”, “Header_Length”, and “HTTPS” strengthen the prediction of “attack traffic”. A high count of reset packets often suggests denial-of-service attacks, as these packets are commonly used to terminate connections abruptly. Additionally, irregular or abnormal header lengths may indicate malicious behavior, such as attempts to obfuscate payloads or exploit protocol vulnerabilities.
- The red-colored features, such as “Variance”, “ack_flag_number”, “urg_count”, and “flow_duration”, represent positive contributions to the prediction. These features suggest that the traffic is less likely to be an attack, reinforcing the classification as benign.

By integrating these observations with our broader understanding of the scenario, we can confidently conclude that the model is both intuitive and reliable in its predictions. For instance, when a flow contains a large number of packets with short time intervals between their delivery, the system becomes more likely to identify potential attacks.

#### 5.5.3. LIME—Local Behavior Analysis

We apply the LIME (Local Interpretable Model-agnostic Explanations) method to interpret the individual predictions made by the XGBoost model. The explanation consists of three main sections and also utilizes a consistent color scheme throughout:

- The estimated prediction for this given instance is displayed on the left.
- The middle part highlights the most significant features, with blue color representing Benign Class (0) and orange color representing Attack Class (1). The relevance of these features is assessed using floating-point numbers.
- The last part displays the actual values of the most relevant variables.

**Figure 13:**
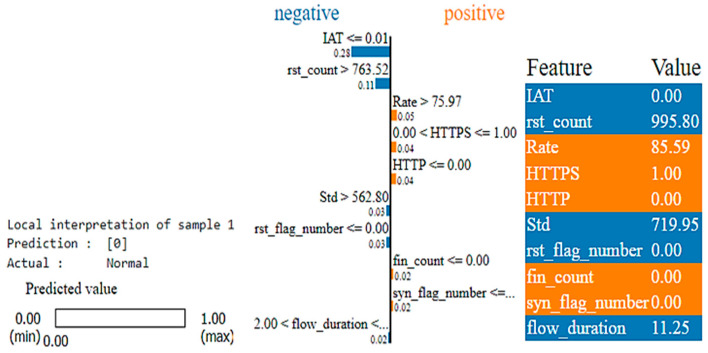
LIME plot for Class 0 (Benign) prediction for Record Sample 1.

For Sample 1, the LIME plot is shown in Figure 13, with the final prediction being “Benign traffic”. The following conclusions can be drawn:

- The features such as “IAT”, “rst_count”, “Std”, “rst_flag_number”, and “flow_duration” are indicated by the blue color, highlighting their negative contribution to the prediction. These features seem to move the classification away from “Benign” traffic.
- Conversely, “Rate”, “HTTPS”, “HTTP”, “fin_count”, and “syn_flag_number” are shown in orange color. These features have a positive influence, supporting the prediction of the traffic as “Benign”. For example, a higher “Rate” (rate of packet transmission) is typically observed in legitimate network traffic, while high “HTTPS” and “HTTP” traffic often point to genuine web traffic. Additionally, values of “fin_count” and “syn_flag_number” within normal ranges suggest regular TCP connection behavior, all contributing to the “Benign” classification.
- The high value of “rst_count” suggests the frequent occurrences of reset packets. This typically indicates network problems or potential malicious activity and, therefore, negatively impacts the classification of traffic as “Benign”.
- The low value of “IAT” indicates that the packet is sent in rapid succession. This value is commonly associated with benign traffic, suggesting regular, uninterrupted communication. However, in this context, the model may associate such frequent transmission patterns with anomalous behavior, thus negatively affecting the prediction for “Benign” traffic. The model likely learned from training data that rapid packet transmission often correlates more with attack traffic than benign traffic, which is why it is marked in blue.

Compared to SHAP, which identifies 20 characteristics, the LIME plot only indicates 10 of the 40 features as relevant and influential. The targeted approach of LIME aids decision-making by emphasizing the key factors that influence the model’s predictions for specific cases. By identifying these important elements, LIME provides a deeper understanding of the logic behind the model’s results, leading to more accurate and informed decisions.

**Figure 14:**
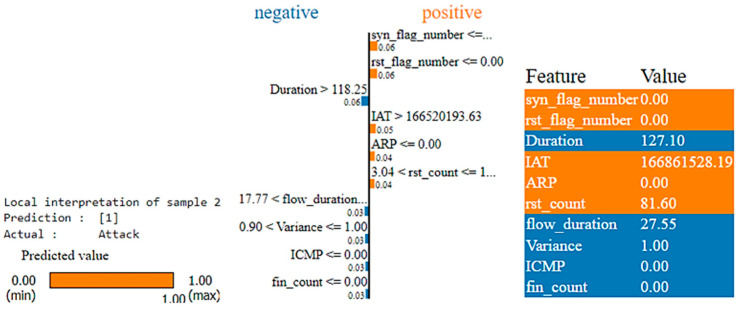
LIME plot for Record Sample 2—predicting Class 1 (Attack).

Figure 14 presents the LIME plot for Test Sample 2, with the prediction of “Attack”. The plot illustrates how different features influence the model’s prediction, with blue features indicating typical traffic behavior and orange features supporting the classification of the traffic as an attack. From this, the following conclusions can be drawn:

- The orange-highlighted features (“syn_flag_number”, “rst_flag_number”, “IAT”, “rst_count”, and “ARP”) contribute positively to the prediction of “Attack” traffic. This suggests that these features increase the probability of classifying the traffic as malicious. For instance, a higher number of SYN and reset flags may indicate disruptive network activity, while a longer inter-arrival time (IAT) and more TCP resets may signal abnormal or suspicious behavior.
- The blue-highlighted features (“flow_duration”, “duration”, “variance”, and “fin_count”) contribute negatively to the prediction of “attack”. This suggests that these features align with typical traffic patterns, where shorter flow durations and low variance are less associated with attack activity. Additionally, a low “fin_count” indicates fewer connection terminations, or that connections are being closed in a predictable, consistent manner, which corresponds to benign traffic behavior.

### 5.6. Cross-Validation of SHAP and LIME Results

The results from LIME and SHAP will be compared in this part, with an emphasis on the lessons learned from two scenarios: Sample 1 (benign traffic) and Sample 2 (attack traffic). This comparison study aims to assess the dependability and consistency of the insights produced by SHAP and LIME in various traffic situations. In order to improve decision-making and model interpretability, we want to make sure that the model’s predictions are clear, reliable, and applicable to a variety of real-world scenarios by looking at both benign and attack traffic. The comparison of LIME and SHAP for both benign and attack traffic is highlighted in the analysis below, showcasing each protocol’s unique insights and consistency across various traffic types.

- Both LIME and SHAP recognize a number of crucial characteristics that help with the classification of benign traffic. Regular web and TCP behavior is reflected in “Rate”, “HTTPS”, “HTTP”, “fin_count”, and “syn_flag_number”, which LIME highlights as favorable signs of benign traffic. In contrast, SHAP shows a wider alignment with typical benign patterns by emphasizing “rst_count”, “HTTPS”, “urg_count”, “Radius”, “ack_flag_number”, “flow_duration”, and “Header_Length” as important for benign traffic.
- LIME identifies “syn_flag_number”, “rst_flag_number”, “IAT”, “ARP”, and “rst_count” as positive indications for attack traffic, indicating the existence of attack patterns. Indicators that indicate deviations from attack characteristics are also identified as negative: “Duration”, “flow_duration”, “Variance”, “ICMP”, and “fin_count”. Similar to this, SHAP employs “IAT”, “rst_count”, “Header_Length”, and “HTTPS” as negative features that support the attack prediction, whereas “Variance”, “ack_flag_number”, “urg_count”, and “flow_duration” are the indicative features that are less likely to be attacked.

In conclusion, both approaches identify “rst_count”, “HTTPS”, and “flow_duration” as influential for benign traffic and highlight important characteristics like “rst_count” and “IAT” for distinguishing between benign and attack traffic. Both offer a complementary perspective on attack traffic, with SHAP emphasizing elements like “IAT”, “rst_count”, “Head-er_Length”, and “HTTPS”, while LIME identifies “syn_flag_number”, “rst_flag_number”, “IAT”, and “ARP” as important. It is also noteworthy that the predictions produced by the SHAP and LIME explanations consistently provide the same class type for a given sample. By forecasting the same class type for a given sample, SHAP and LIME align, demonstrating the consistency and dependability of the model’s behavior and bolstering confidence in its interpretability and decision-making process.

### 5.7. Analysis of XGBoost Predictions Using XAI Results

To confirm that the outcomes from each of these techniques—XGBoost, SHAP, and LIME—are consistent, the study entails a number of crucial procedures. First, we compare the feature importance rankings provided by XGBoost, SHAP, and LIME to identify both common and distinct features across the methods. This comparison allows us to understand how each technique interprets and prioritizes features in relation to the model’s predictions. Next, we focus on the features that are consistently recognized as important across all three methods—XGBoost, SHAP, and LIME. This consistency is essential for validating that these features are genuinely relevant and robust, particularly in addressing the intricate security challenges expected in 6G environments. We also cross-verify these key features across the methods to confirm their alignment and ensure the model’s decision-making process remains consistent, a crucial step in achieving the transparency and reliability necessary for autonomous and complex 6G systems. Finally, we examine how these significant features affect the model’s predictions and confirm that their impact is consistent across the different methods. This thorough analysis ensures that the model’s predictions are interpretable, trustworthy, and capable of supporting critical security decisions in the highly dynamic and intelligent landscape of 6G networks. The following analysis can be made:

- “rst_count” and “IAT” are highlighted by both XGBoost and SHAP as being extremely important features for the model’s prediction.
- It is observed that the feature “Protocol Type” has the least impact. This can be a result of the information it offers being redundant or overlapping with other, more significant features. For example, flags such as “ack_flag_number”, “syn_count”, “fin_count”, and “urg_count”, which are highlighted as influential features, offer detailed insights into the behavior of TCP connections, often implicitly indicating the protocol in use, rendering “Protocol Type” feature as somewhat redundant. Moreover, features like “Rate” and “Header_Length” can indirectly capture protocol-related characteristics, while the inclusion of HTTPS/HTTP features further diminishes the standalone importance of “Protocol Type”. Since these other features collectively capture the key characteristics that “Protocol Type” would convey, its contribution to the model’s predictions becomes less significant.
- Both approaches consistently rank variables including “flow_duration”, “HTTPS”, “ack_flag_number”, “Variance”, “Header_Length”, “Rate”, “Std”, “Max”, “Magnitude”, “Radius”, “Duration”, and “Covariance” highly, demonstrating their significance. The trustworthiness of these features in the model is further supported by the overlap in feature priority rankings between XGBoost and SHAP. Additionally, by comparing the feature importance results from XGBoost and SHAP, we can be sure that both approaches concur on the key elements that influence the model’s predictions, improving the decision-making process’s dependability and transparency.
- Both approaches emphasize “rst_count”, “IAT”, and “flow_duration” as important aspects when comparing the predictions of the XGBoost model with the outcomes of LIME, highlighting their significance in the model’s decision-making process. This widespread acknowledgment highlights the consistent function of these variables in traffic type prediction, whether using the global model perspective of XGBoost or the localized explanations of LIME.

In conclusion, essential characteristics including “rst_count”, “IAT”, “flow_duration”, “Rate”, and “Header_Length” are consistently identified as significant in the comparison of XGBoost, SHAP, and LIME. This agreement amongst the approaches emphasizes how reliable and strong these traits are in influencing the model’s predictions, underscoring their critical function in identifying and evaluating IoT threats.

### 5.8. Recursive Feature Elimination

**Figure 15:**
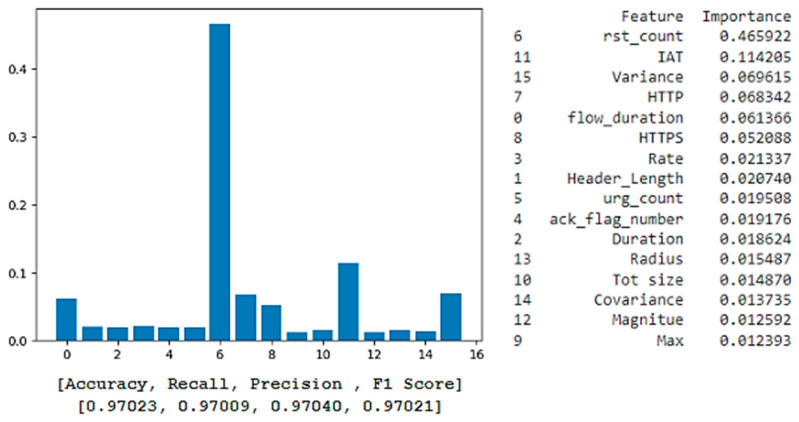
XGBoost feature importance plot and feature importance score list—after gathering insights from XAI.

This section describes a methodical way to integrate XGBoost with XAI approaches for feature selection and evaluation. This approach efficiently improves prediction performance and addresses the advanced demands of 6G networks by enhancing accuracy and identifying critical features. We start by examining XGBoost insights and removing features that have a zero score, which suggests that they have little bearing on predictions. After that, we identify both important and unimportant features using XAI algorithms. As shown in Figure 15, by iteratively improving the XGBoost model with these chosen features, we were able to significantly increase accuracy from 95.59% to 97.02%. Feature 6 (rst_count) and Feature 12 (IAT), which were previously recognized as high-ranking features, are confirmed to have the most influence on forecasting the output by the feature importance score list. This highlights that prioritizing the most relevant features can substantially enhance model performance, delivering reliable and accurate predictions essential for addressing the sophisticated, data-driven, and autonomous operations anticipated in 6G networks.

## 6. Conclusions

Fostering a safer and more secure emerging IoT–6G ecosystem that is expected to host applications essential to humanity requires ensuring transparency and confidence in AI-powered systems. We run the risk of exposing users and vital infrastructure to risks and vulnerabilities that could jeopardize social cohesion and financial stability if we do not have a comprehensive understanding of how predictive models in AI-based systems function. Understanding and deciphering the inner workings of AI models is not only advantageous but also essential to preserving the integrity, robustness, and dependability of the globalized society we are building.

In this study, we evaluate various machine learning models, including Random Forest, Logistic Regression, KNN, and XGBoost with the CICIoT 2023 dataset. This dataset encompasses both live and normal traffic generated by a diverse range of IoT devices. Starting with the dataset that initially exhibits class imbalance, we achieve good enhancement of the reliability of predictions by employing the SMOTE technique. This technique balances all the subcategories of attacks within the main classes and removes biases to improve predictions. The models are then evaluated based on prediction accuracy and feature importance to identify the one with the most reliable performance. Once the best-performing model is identified, we delve deeper into its decision-making process using explainable AI (XAI) techniques, such as SHAP and LIME. We establish that the predictions of the XGBoost algorithm are in agreement with the explanations given by SHAP and LIME by examining both attack and benign dataset samples.

The evaluation conducted using the proposed framework ensures consistency, thereby validating the effectiveness of the target model. Additionally, we verify the coherence of the explanations offered by SHAP and LIME for the same data set by cross-checking them. By removing less important features—especially those with zero importance scores—and giving priority to important features found by XAI approaches, recursive feature removal is used to improve detection accuracy using these insights. The accuracy of this improved method is 97%, which is significantly higher than the 95% accuracy of XGBoost without explainability techniques.

Our research highlights the critical significance of integrating AI models with explainable AI (XAI) approaches to improve decision-making, uncover hidden patterns, and address the difficulties presented by changing 6G settings. In addition to strengthening threat detection and network security, our collaboration gets us ready to handle the challenges brought on by the increasing integration of IoT systems and cutting-edge wireless technologies. By providing deeper insights into model behavior and improving detection accuracy, our research facilitates the implementation of precise security measures to effectively counter cyber threats, ultimately ensuring a more secure and robust IoT–6G ecosystem.

In future research, we plan to enhance experiments using 6G simulations or datasets tailored to 6G-specific characteristics. We will also analyze the impact of feature redundancy and correlation on performance by comparing methods like RFE and PCA to validate the framework’s robustness. Furthermore, to ensure practical applicability in 6G–IoT environments, such as telemedicine or industrial automation, we will evaluate the model’s latency and computational complexity, focusing on maintaining high performance in low-latency, time-sensitive scenarios.

## Figures and Tables

**Table 1:** Description of features in the CICIoT dataset.

Feature Num.	Feature	Description
0.	flow_duration	Time between the first and the last packet
1.	Header_Length	Header length
2.	Protocol type	TCP, IP, UDP, ICMP, IGMP, Unknown (Integers)
3.	Duration	Time to live (ttl)
4.	Rate	Pace at which packets are transmitted within a flow
5.	Srate	Rate at which packets are sent out within a flow
6.	fin_flag_number	Value of fin flag
7.	syn_flag_number	Value of syn flag
8.	rst_flag_number	Value of rst flag
9.	psh_flag_number	Value of psh flag
10.	ack_flag_number	Value of ack flag
11.	ack_count	Quantity of packets with ack_count in a network flow.
12.	syn_count	Count of packets in the same flow with syn flag set
13.	fin_count	Count of packets with the FIN flag in a network flow
14.	urg_count	Quantity of packets with urg flag in a network flow
15.	rst_count	Count of packets where the RST flag is enabled
16.	HTTP	Identifying if HTTP is the application layer protocol
17.	HTTPS	Identifying if HTTPS is the application layer protocol
18.	DNS	Identifying if DNS is the application layer protocol
19.	SSH	Identifying if SSH is the application layer protocol
20.	TCP	Identifying if TCP is the transport layer protocol
21.	UDP	Identifying if UDP is the transport layer protocol
22.	ARP	Identifying if ARP is the link layer protocol
23.	ICMP	Identifying if ICMP is the network layer protocol
24.	IPv	Identifying if IP is the network layer protocol
25.	LLC	Identifying if LLC is the link layer protocol
26.	Tot sum	Total packets in a flow
27.	Min	Min packet length
28.	Max	Max packet length
29.	AVG	Avg. packet length
30.	Std	Deviation from the mean of packet length within a flow
31.	Tot size	Packet’s length
32.	IAT	Time difference between the consecutive packets
33.	Number	Count of packets
34.	Magnitude	Square root of the total average packet lengths inside the flow, including incoming and outgoing
35.	Radius	Square root of the total variation in the inbound and outgoing packet lengths throughout the flow
36.	Covariance	Connection between the outgoing and incoming packet lengths
37.	Variance	Disparity in packet lengths between incoming and outgoing packets within a given flow
38.	Weight	Ratio of incoming packets to outgoing packets within a flow
39.	DHCP	Identifying if DHCP is the application layer protocol

**Table 2:** Categorization of dataset.

Sl. Num	Class Categories	Instances	Overall Count
1	DDoS	72,776	97,624
2	DOS	17,392	
3	Mirai	5525	
4	Spoofing	1034	
5	Recon	817	
6	Web	52	
7	Brute force	28	
8	Benign	2376	2376

**Table 3:** Performance evaluation metric.

Model	Accuracy	Precision	Recall	F1-Score
Logistic regression	70.47%	0.7058	0.7037	0.7036
K-nearest neighbor	87.70%	0.8780	0.8727	0.8739
XGBoost	95.23%	0.9525	0.9524	0.9523
Random forest	94.40%	0.9448	0.9432	0.9439
CNN	65.36%	0.6536	0.6536	0.6536

**Table 4:** List of best features identified by XGBoost and Random Forest.

Influential Features in Model Prediction	XGBoost	Random Forest
1st Top Feature	rst_count	rst_count
2nd Top Feature	IAT	IAT
3rd Top Feature	variance	Header_Length
4th Top Feature	flow_duration	flow_duration
5th Top Feature	HTTPS	HTTP
6th Top Feature	urg_count	HTTPS

## Data Availability

Data is contained within the article. The data supporting the findings of this study, including the figures and results, are presented within the paper. The code used for the analysis is available upon request.
