# Correction: Kaur, N.; Gupta, L. Securing the 6G–IoT Environment: A Framework for Enhancing Transparency in Artificial Intelligence Decision-Making Through Explainable Artificial Intelligence. *Sensors* 2025, *25*, 854

**DOI:** 10.3390/s26010336

**Published:** 2026-01-05

**Authors:** Navneet Kaur, Lav Gupta

**Affiliations:** Department of Computer Science, University of Missouri, St. Louis, MO 63121, USA; nk62v@umsystem.edu

## Text Correction

In the original publication [1], the term “assault” was used in a few instances. As this term is not commonly used, it has been replaced with “attack” to improve clarity. The corrected content appears below.

1. Introduction

Paragraph 2: “denial-of-service assaults” is replaced by “denial-of-service attacks”.

5.4. Model Selection for XAI Technique Application

Paragraph 5: “DDoS assault” and “Brute-Force assault” are replaced by “DDoS attack” and “Brute-Force attack”.

Paragraph 6: “DoS assault” is replaced by “DoS attack”.

5.5.2. SHAP—Local Behavior Analysis

Paragraph 4: “possible assaults” is replaced by “possible attacks”.

Paragraph 5: “DoS assaults” is replaced by “DoS attacks”.

The authors confirm that the scientific conclusions, experimental results and overall findings of the study remain unaffected. This correction was approved by the Academic Editor. The original publication has also been updated.
